# How to minimize pain with local anesthesia and improve patient experiences: a review

**DOI:** 10.1016/j.abd.2026.501366

**Published:** 2026-05-09

**Authors:** Umer Nadir, Isadora Rinaldo Scaburi, Felipe Bochnia Cerci, George Michael Jeha, Stanislav Nickolaevich Tolkachjov

**Affiliations:** a Epiphany Dermatology, Dallas, TX, United States; b Department of Dermatology, Texas A&M School of Medicine, Dallas, TX, United States; c Division of Dermatology, Baylor University Medical Center, Dallas, TX, United States; d Division of Dermatology, Hospital Universitário Evangélico Mackenzie, Curitiba, PR, Brazil; e Clínica Cepelle, Curitiba, PR, Brazil; f Department of Dermatology, University of Texas at Southwestern, Dallas, TX, United States

**Keywords:** Anesthesia, local, Anesthetic, Injections, Lidocaine, Pain, Review

## Abstract

**Background:**

Local anesthesia is essential in common procedures in dermatology, but injection and infiltration of anesthetic often cause significant pain, reducing patient comfort and satisfaction.

**Objective:**

To review evidence-based techniques to reduce pain associated with local anesthetic injection and infiltration.

**Methods and materials:**

A narrative review of the literature was performed, and techniques were summarized for clinicians to reference.

**Results:**

Technical methods to reduce pain include using smaller-gauge needles (30–33G), smaller syringes (1‒3 mL), buffering lidocaine with sodium bicarbonate, inserting the needle at 90 degrees, slow infusion, subcutaneous delivery, and pore-guided injection. Adjuncts such as warming anesthetic, vibration, ice, music, and verbal distraction (“talkesthesia”) also reduce patient perception of pain.

**Study limitations:**

Limitations include heterogeneity in study designs, outcomes measures, and clinical contexts across the included literature, precluding quantitative synthesis or direct comparison of individual techniques. Additionally, several recommendations are based on expert opinion or limited evidence.

**Conclusion:**

A multimodal approach using both technical refinements and adjunctive measures can make local anesthetic administration less painful. Dermatologists may routinely implement these strategies to improve patient experience and satisfaction.

## Introduction

Local anesthesia is ubiquitous in medical practice, commonly used for a wide range of procedures, including biopsies, cauterization, excision, and surgical wound reconstruction.^1^ The injection of local anesthetic is often considered the most painful aspect of these procedures and optimizing injection technique is essential for surgeons to ensure patient comfort and safety.^2^ Moreover, minimizing patient discomfort during the injection of local anesthesia has been shown in the literature to deliver positive patient-reported outcomes.^3^

Patients are often warned about the two sources of discomfort associated with local anesthetic infiltration. The first source of discomfort is caused by a needle penetrating the skin, which provokes a sharp, brief pain. The second source of discomfort is through infiltration of the solution, which activates cutaneous nociceptors through both mechanical stretching of the tissues and exposure to the anesthetic’s acidic pH.

Fortunately, there are proven strategies to make local anesthesia nearly painless. The purpose of this article is to gather, discuss, and illustrate techniques in reducing pain with local anesthesia and to encourage their routine adoption with a focus on patient comfort and excellence in care.

## Preparation for injection

### Needle gauge

Smaller needles (larger gauges) are associated with significantly decreased intensity of pain during insertion.^4^ This has been confirmed by several studies in the literature as these smaller needles require less force to penetrate the skin, come into contact with fewer nociceptors, and decrease the rate of anesthetic injection.^5^, ^6^ Additionally, the smaller diameter also allows for easier penetration in areas of increased skin thickness, such as the palm of the hand or the sole of the foot.^3^ These findings are consistent with clinical practice, where smaller-gauge needles are preferred for procedures requiring superficial injections, such as those on the face, due to the decreased pain. A recommendation from the authors for anesthesia on the face includes the use of a 30G or 33G needle due to greater precision and less discomfort during application, especially in more sensitive areas.

### Syringe size

The intensity of pain during the injection of local anesthetic is also influenced by syringe size, even when needle sizes remain constant.^7^, ^8^ It is hypothesized that smaller syringes (1 mL) are associated with less pain, likely because they require less mechanical force to inject the anesthetic and allow for a smoother, controlled infiltration of fluid into the tissues. This is exemplified by a “split-scalp” study of 20 patients undergoing hair restoration surgery. Lidocaine with epinephrine (1:100,000) was delivered using a 30G needle with either a 1 mL or 3 mL syringe. The results showed that 1 mL syringes caused significantly less pain than the 3 mL syringes.^7^ A recommendation from the authors is to start with 1 mL syringe and transition to a 3 mL syringe to optimize efficiency without compromising patient comfort. If more control is required in the hands of the injector, consider filling the 1 mL syringe halfway. In anatomically sensitive regions, including the nasal tip and perioral area of patients, the 1 mL syringe can be used alone.

### Buffering

Buffering of lidocaine with sodium bicarbonate has been shown to reduce the burning pain associated with infiltration through increasing the pH of the acidic anesthetic formulations. Studies have shown that a 1:10 ratio of 8.4% sodium bicarbonate to 1% lidocaine with 1:100,000 epinephrine effectively minimizes discomfort without compromising anesthetic efficacy.^6^, ^9^ As stated in the joint position statement by the American Academy of Dermatology (AAD), American College of Mohs Surgery, American Society For Dermatologic Surgery, and American Society For Mohs Surgery it is recommended that physicians and their clinic staff meet or exceed the safety standards of the U.S. Pharmacopeial Convention and the FDA Insanitary Conditions Guidance as buffering is subject to compounding regulations at the federal level and possibly the state level.^10^ Furthermore, buffered lidocaine begins to lose its vasoconstrictive efficacy after seven days and should be administered by then, with appropriate labelling with expiration dates.^11^, ^12^

## Injection technique

### Needle insertion angle

When the needle is inserted into the skin, nociceptors in the dermis are directly activated.^5^, ^6^ By penetrating the skin at a 90-degree angle as compared to a 45-degree angle, the needle passes through fewer nociceptors and thus causes less pain.^13^ In a clinical trial comparing pain during application of infiltrative local anesthesia using lidocaine, there was a statistically significant difference in pain levels between needle insertion angles. Injections performed at 90-degrees had a median pain level of 2.0 (IQR 2.0‒3.0) vs. injections performed at 45-degrees had a median pain level of 3.0 (IQR 2.0‒4.0, p = 0.002). Furthermore, patients independently reported that the 45-degree injection was more painful than the 90-degree injection. These results indicate that the 90-degree insertion angle provides a less painful experience to patients.^14^ It is important to note that once the needle has been inserted and a small amount of local anesthesia has been injected, the needle can be “reangulated” so that the local anesthesia can be better dispersed throughout the surrounding area or “walked”. The authors recommend that when inserting at 90 degrees is not feasible or is suboptimal, such as in the case of eyelid skin, fine motor control with counter tension applied with the opposite hand and injection at an angle would be appropriate. Injection at an angle may allow better support and stability in these specific cases due to better hand positioning, and allows clinicians to “walk” the anesthesia in the direction they wish.

### Rate of anesthetic infusion

Another factor influencing pain during infiltration of an anesthetic is the activation of nociceptors responding to rapid tissue stretch and distention.^3^ By reducing the rate of injection, an anesthetic has the ability to diffuse and block nerve transduction of stimulated fibers, functionally eliminating “the burn” classically associated with local anesthesia.^3^, ^6^, ^8^, ^15^, ^16^, ^17^, ^18^ Furthermore, rapid injection has been associated with increased levels of pain with injection and consequently decreased patient satisfaction with their procedure.^19^, ^20^ A recommendation from the authors is that slow infusion of anesthetic is a major contributor to reducing pain when injecting local anesthesia. Furthermore, a recommendation from the authors is to meticulously clear the syringe and syringe of any air and depress the plunger while advancing the needle into the skin, as the local anesthetic would then be injected the instant the entire opening of the needle is below the skin surface.

### Injection plane

**Figure 1:**
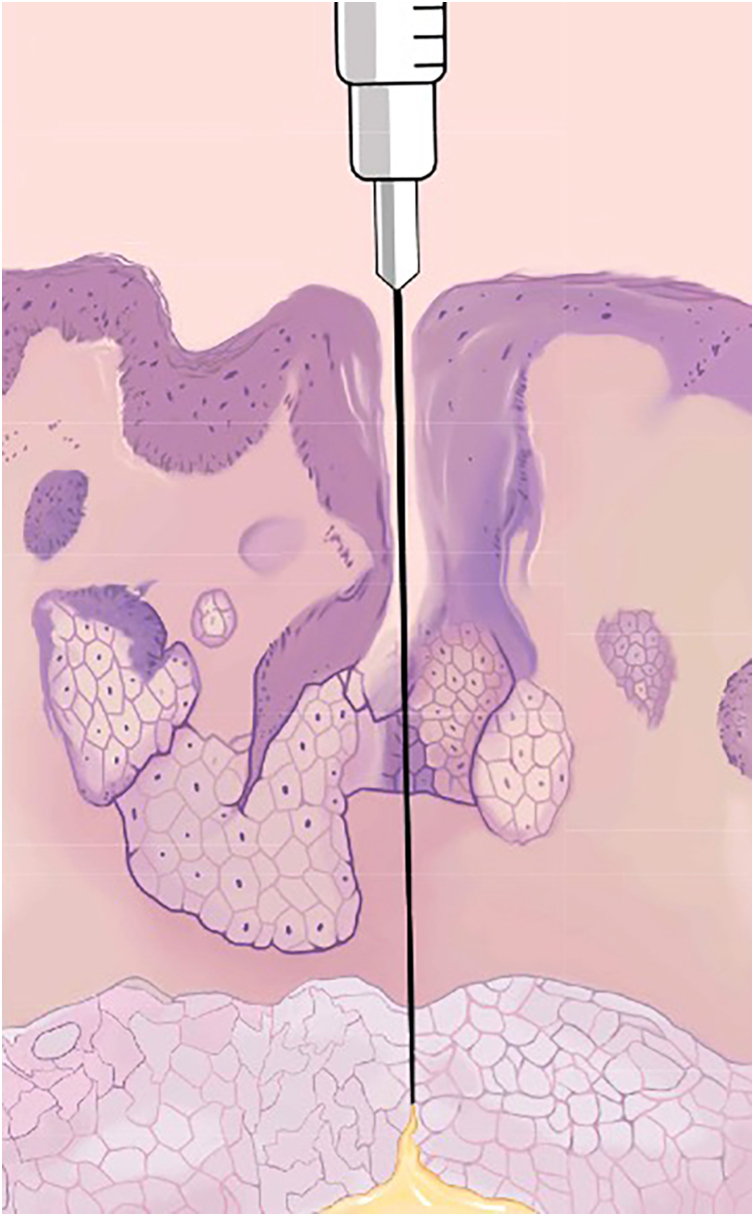
Injection plane and pore-guided injection.

When considering injection depth, subcutaneous injections cause less pain than intradermal injections while achieving the same end-goal analgesic effect.^6^ However, intradermal injections induce anesthesia more quickly than subcutaneous injections. As such, it may be appropriate to raise an intradermal wheal, withdraw, then reintroduce the needle at the numbed site toward the subcutaneous plane (Fig. 1). By withdrawing and reintroducing the needle through the intradermal wheal, it is possible to substantially reduce a patient’s burden of pain from needle sticks.^3^, ^13^ Furthermore, subcutaneous injections also treat the nerve roots supplying the dermis, providing analgesia without having to anesthetize the dermal layer specifically, which would cause a patient much more pain.^6^ It is notable that these larger nerve roots take a longer time to numb.

### Injection volume

**Figure 2:**
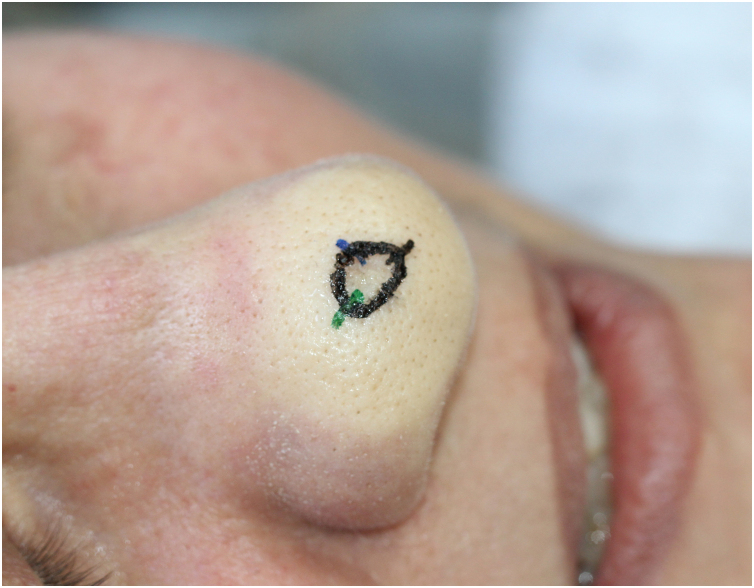
Edema endpoint.

Anesthetic should be administered with an endpoint of forming visible edema in the procedural area. No robust comparative studies between volumes of anesthetics have been published so far in the literature. Larger volumes have been observed to virtually eliminate the risk of pain during interventions and studies observing peak serum concentrations of lidocaine during Mohs micrographic surgery show safety and efficacy in large volumes during the procedure.^18^ Furthermore, increased volumes in the procedural area contribute to mechanical vasoconstriction, which helps control bleeding (Fig. 2). It is paramount to highlight that anesthetic should be applied to areas that also extend beyond incision margins, since undermining, sutures, and the use of thermal cautery can cause pain in adjacent regions.

### Use of warmed solution

A systematic review and meta-analysis demonstrated that solutions warmed to 37 °C have been shown to reduce pain levels during the administration of local anesthesia compared to non-warmed solutions.^21^, ^22^, ^23^ Furthermore, studies using warmed tumescent solutions have also shown a significant reduction in pain compared to room-temperature solutions.^24^, ^25^

### “Pore-guided” injection

**Figure 3:**
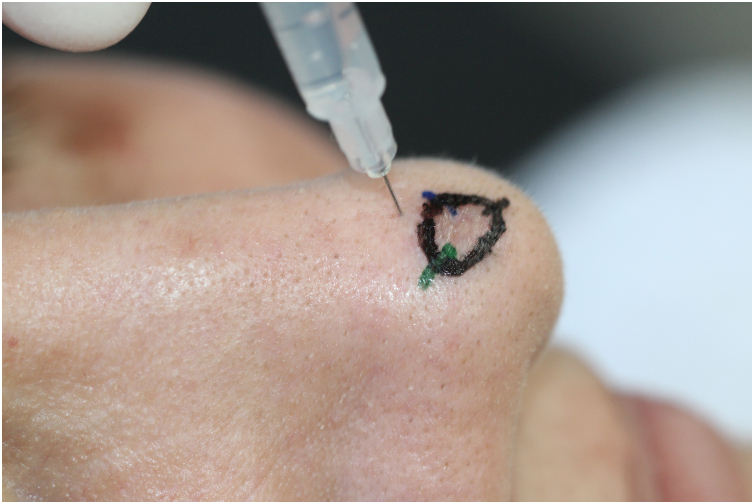
Needle Insertion Angle. It is recommended to first inject the proximal part of the nasal tip because it will “block” the distal portion.

A recommendation from the authors is to consider introducing the needle through a pore, which acts as an isolated tunnel or portal “shortcut” for the needle (Fig. 3).^41^ By doing this, it is possible to reach deeper planes with less trauma in the dermal layers, especially in the reticular dermis, which hosts more sensitive structures. However, systematic reviews and further studies to robustly prove the efficacy of this technique are lacking.

### “Walking”

The authors recommend that when numbing a larger area, after the initial syringe is used, insert subsequent needles at the margin of the already numbed area so the patient does not feel each poke. Furthermore, angling the needle toward the margin and allowing the fluid wave to move forward advances the anesthetized area while causing less discomfort to the patient. Examples of utility for this technique include numbing the tarsal plate or the palmar surface of the fingertip. For the tarsal plate, inject the eyelid and allow the fluid wave to infiltrate and numb the tarsus. Similarly, start with the thinner skin on the lateral or dorsal finger and “walk” the anesthetic to the palmar surface. In the experience of the authors, on the nose, areas with subcutaneous adiposity are easier to start with and less painful for patients. Therefore, if the surgical site is near an area with subcutaneous adiposity, start within the adiposity and “walk” the anesthesia to the target site.

## Adjunct tools

### Background music

Various articles in the literature have studied background music and its beneficial effects on pain associated with local anesthesia. Evidence is varied and depends on the specific clinical context, but most notably, a clinical trial studying the intervention of classical music vs silence during anesthetic injection showed significantly decreased pain and anxiety scores after exposure to classical music.^26^ This study is limited in its subjectivity, but as pain is a subjective measure, this study points to music as a simple, safe, and effective intervention in surgical practice.

### Cooling the skin

Cooling the skin prior to infiltration of local anesthetic has been shown to significantly reduce pain related to the procedure.^27^ A randomized controlled study evaluated the application of ice before local anesthesia injections for simple laceration repair and found a significant reduction in pain perceived by patients.^28^ Another study compared the use of ice vs topical anesthetics during botulinum toxin injections and concluded that ice provided pain relief comparable to the topical anesthetic, suggesting that ice may be a convenient option due to its shorter application time.^29^ However, the guidelines of the American Academy of Dermatology (AAD) indicate that, although ice is safe, data on its effectiveness in reducing infiltration pain from local anesthesia are limited and contradictory.^1^

### Local vibration

Evidence suggests that stimulation of Meissner’s and Pacinian corpuscles through the use of a vibratory device substantially reduces the sensation of pain during the injection of local anesthesia.^30^, ^31^ The reduction of pain can be explained by neurophysiologic mechanisms, including gate control theory and other complex interactions within the nervous system that mask pain with injection and subsequent infiltration. Gate control theory suggests that the activation of nerve fibers that conduct non-noxious stimuli, such as Aβ fibers, can inhibit the transmission of pain signals to the central nervous system, thus reducing the perception of pain.^32^, ^33^, ^34^ Furthermore, vibration has been shown to induce kinesthetic illusions that also contribute to analgesia.^35^ In a clinical trial evaluating the use of a vibratory device to mitigate pain during local anesthesia, the use of the device resulted in a 40% significant reduction in pain during injection and infiltration.^36^ Therefore, the low side effect profile and ease of use for this technique warrant consideration for its inclusion in daily practice. A recommendation from the authors, in consideration of efficiency, is manual vibration performed with the practitioner’s own fingers. Although this may provide less intense vibration compared to a device, this technique helps modulate the patient’s sensory perception and reduce discomfort without the need for an additional device. Mechanical stimulation prior to injection by physicians has been studied previously and has been shown to have a significantly reduced patient’s perception of pain.^37^, ^38^

### “Talkesthesia”

Guidelines set forth by the AAD state that verbal distraction is a recommended technique to reduce pain during local anesthetic infiltration.^1^ This recommendation was based on expert opinion, and the authors encourage engaging patients by discussing topics that generally appeal to most people, such as hobbies, cooking, grandchildren (for elderly patients), movies, books, music, etc. One study demonstrated that the use of engaging, kind, and reassuring words during the administration of local anesthetics can improve the perception of pain and increase comfort compared to the use of negative words.^39^ It is important to consider this technique as part of a broader set of strategies to minimize pain with anesthetic infiltration.

### Topical anesthetics

Studies evaluating the use of topical anesthetics prior to injection of local anesthetic show that they may be effective in some patient cases, but the long period required for them to remain on the skin for their effect to take place is a significant disadvantage to efficiency. Furthermore, a recent randomized controlled trial found no significant difference in pain reduction during administration of local anesthesia on the head and neck region when comparing the use of a 2.5% lidocaine and 2.5% prilocaine emulsion, ethyl chloride “cold spray”, and a topical control.^40^

### Asking patients to close their eyes

The authors recommend that clinicians either cover their patients’ eyes or ask their patients to close their eyes during local anesthetic administration. In the authors’ experience, patients become more nervous when watching. Furthermore, this provides added protection to patients as local anesthetics can squirt out of a pore, or the needle may separate from the syringe in some cases.

## Conclusion

Several techniques described in this review have been clinically proven to reduce pain during the injection and infiltration of local anesthesia. Specifically, smaller needle gauges and syringes, buffering of acidic solution, 90-degree injection angle, slow infusion, subcutaneous and larger volume infiltration, and warmed solution have been proven in the literature. Adjuncts to injection and infiltration that have also been clinically proven include background music, cooling of the skin, local vibration, “talkesthesia”, and topical anesthetics. By implementing a combination of these techniques, it may be entirely possible to make the process of local anesthesia virtually painless to the patient (Supplementary Video 1). Surgeons are commonly assessed by patient-reported outcomes such as mitigation of pain during procedures and as such, surgeons should make all efforts to ensure a patient’s experience is as painless as possible. Furthermore, in many clinics, a local anesthetic is administered by ancillary staff. It may be beneficial to formally instruct those administering local anesthetics or consider developing training materials on the concepts and techniques mentioned in this manuscript. The authors provide several recommendations commonly used by colleagues in addition to those proven in the literature (Table 1, Table 2). These include the use of a 30G or 33G needle on areas of the face, starting with a 1 mL syringe, introducing the needle through a pore, manual vibration with the practitioner’s own fingers, “walking” anesthetic towards target sites, engaging patients by discussing topics that generally appeal to most people, and covering/closing patients’ eyes. It is important to prioritize many factors when implementing these recommendations, including the anecdotal nature of some recommendations, differences in clinic operations, regulations, the procedure being performed, and anatomic location. Future research should focus on comparing these techniques in randomized controlled trials to ascertain the optimal course of local anesthesia for these patients.

**Table 1:** Summary of recommendations for minimizing pain with injection and infiltration of local anesthesia.

Technique	Recommendation
Small Needle Gauge	Consider a 30G or 33G needle.
Small Initial Syringe Size	Consider starting with a 1 mL syringe, then transition to a 3 mL syringe.
Buffering	Consider buffering lidocaine with sodium bicarbonate at a 1:10 ratio of 8.4% sodium bicarbonate to 1% lidocaine with 1:100,000 epinephrine.
90-degree Needle Insertion Angle	Consider penetrating the skin at a 90-degree angle instead of a 45-degree angle.
	When inserting at 90-degree is not feasible, fine motor control with counter tension applied with the opposite hand and injection at an angle would be appropriate
Rate of Anesthetic Infusion	Consider slow infusion of anesthetic during infiltration.
	Clear any air from the syringe and needle, and depress the plunger while inserting the needle into the skin
Subcutaneous Injection Plane	Consider aiming to inject subcutaneously rather than intradermally.
	Consider raising a wheal, withdrawing the needle, then re-introducing the needle at the site of the wheal
Large Injection Volume	Consider forming visible edema in procedural area during infiltration. Apply to surrounding areas, beyond incision margins.
Use of Warmed Solution	Consider warming solutions to 37 °C.
“Pore-guided” injection	Consider introducing the needle through a pore.
“Walking”	After initial infiltration, consider inserting subsequent needles at the site of the already numbed area.
	Consider angling the needle toward the margin and allowing the fluid wave to advance the anesthetized area.

**Table 2:** Adjunct tools summary.

Technique	Recommendation
Background music	Consider playing music that the patient enjoys.
Cooling the Skin	Consider application of ice prior to injection.
Local vibration	Consider manual vibration performed by the practitioner’s own fingers during infiltration.
“Talkesthesia”	Consider engaging patients by discussing topics that generally appeal to most people such as hobbies, cooking, grandchildren (for elderly patients), movies, books, music, etc.
Topical Anesthetics	Consider combining other options.
Asking patients to close their eyes	Consider covering the patient’s eyes or ask patients to close their eyes.

## ORCID ID

Umer Nadir: 0000-0002-2140-9302

Isadora Rinaldo Scaburi: 0000-0002-4719-1989

Felipe Bochnia Cerci: 0000-0001-9605-0798

George Michael Jeha: 0000-0003-0442-2836

## Abbreviations

mL, Milliliter; G, Gauge; IQR, Interquartile Range; °C, Degrees Celsius; AAD, American Academy of Dermatology.

## Research data availability

The entire dataset supporting the results of this study was published in this article.

## Financial support

None declared.

## Authors' contributions

Umer Nadir: Contributed to study conception, data acquisition, analysis, and interpretation, and drafted substantial portions of the manuscript, reviewed and approved the final manuscript and agreed to be accountable for all aspects of the work.

Isadora Rinaldo Scaburi: Contributed to data interpretation and drafted the original version of the manuscript, reviewed and approved the final manuscript and agreed to be accountable for all aspects of the work.

Felipe Bochnia Cerci: Contributed to the conceptual framework and provided critical intellectual input to the study design and content, reviewed and approved the final manuscript and agreed to be accountable for all aspects of the work.

George Michael Jeha: Contributed to critical revision of the manuscript for important intellectual content, reviewed and approved the final manuscript and agreed to be accountable for all aspects of the work.

Stanislav Nickolaevich Tolkachjov: Supervised the study, contributed to study design and interpretation of data, provided critical revision and final approval of the manuscript, reviewed and approved the final manuscript and agree to be accountable for all aspects of the work.

## Conflicts of interest

Stanislav Nickolaevich Tolkachjov is an investigator/speaker for Bioventus, Kerecis, Boehringer Ingelheim, CASTLE, and Regeneron. Other authors have no relevant conflict of interest to declare.
